# Changes in oxidized LDL during a half marathon in athletes with spinal cord injuries

**DOI:** 10.1038/scsandc.2017.15

**Published:** 2017-05-11

**Authors:** Toshihito Mitsui, Tomoyuki Ito, Yusuke Sasaki, Takashi Kawasaki, Takeshi Nakamura, Yukihide Nishimura, Tatsuru Ibusuki, Yukiharu Higuchi, Sayoko Hosoe, Fumiaki Ito, Fumihiro Tajima

**Affiliations:** 1 Department of Rehabilitation Medicine and Sports Medical Center, Wakayama Medical University School of Medicine, Wakayama City, Japan; 2 Faculty of Sports Science, Nihon Fukushi University, Mihama, Aichi, Japan; 3 Institute of Health Sciences, Sunstar Inc., Osaka, Japan

**Keywords:** Nutrition, Fat metabolism

## Abstract

**Introduction:**

We reported previously that exercise significantly increases plasma adrenaline and oxidized low-density lipoprotein (oxLDL) in healthy subjects but not in persons with spinal cord injury (SCI). Since oxLDL and adrenaline levels are associated with oxidant/antioxidant balance, and exercise training elicits production of reactive oxygen species, we elucidated the effects of exercise on adrenaline, oxidant/antioxidant balance and oxLDL in individuals with SCI.

**Case Presentation:**

Eight subjects with cervical spinal cord injury (CSCI) and nine subjects with lower lesion of SCI (lower SCI (LSCI)) participated in a wheelchair half marathon race, and blood samples were collected before (pre), immediately after (post) and 1 h after the race (post 1 h). The blood samples were used to determine adrenaline, derivatives of reactive oxygen metabolites (d-ROMs) and biological antioxidant potential (BAP), both as markers for oxidant/antioxidant balance.

**Discussion:**

Pre-serum oxLDL levels were 147.2±8.1 and 97.0±10.4 U l^−1^ (mean±s.e.m.) in LCSI and CSCI subjects, respectively, and remained stable throughout the study. Adrenaline levels were higher in LSCI athletes than in CSCI athletes, especially post half marathon. Serum d-ROMs level did not change between pre and post in both groups. The mean BAP was significantly higher in LSCI than in CSCI subjects (2574±94.6 vs 2118±94.6 μmol l^−1^) at post, whereas the oxidative stress index (d-ROMs/BAP) was similar in the two groups throughout the study. In conclusion, exercise did not increase the d-ROMs or d-ROMs/BAP ratio in CSCI and LSCI subjects. The lack of increase in the plasma oxLDL level in SCI subjects was not due to the lack of response of adrenaline to exercise.

## Introduction

Oxidized low-density lipoprotein (oxLDL) enhances the formation of atherosclerotic plaques and increases the risk of coronary artery disease, type 2 diabetes mellitus and atherosclerosis. Physical exercise is known to reduce the risk of these diseases, causing oxidative stress that eventually induces an antioxidative response, which reduces the levels of oxidized lipids, proteins and DNA. Chronic aerobic exercise reduces LDL oxidation,^1,2^ but acute strenuous exercise is often followed by a significant increase in oxLDL.^3^ Furthermore, regular exercise has been reported to reduce oxLDL levels in patients with heart diseases, hypertension, dyslipidemia and type 2 diabetes mellitus.^4,5^

Reactive oxygen species (ROS) trigger the formation of oxLDL. ROS are a group of compounds endowed with high reactivity and short half-life based on their tendency to give or receive electrons to attain stability.^6^ Regular physical exercise has many health benefits, but intense and prolonged exercise induces excessive formation of ROS in various tissues, such as skeletal muscles and liver, leading to a shift in redox balance in favor of oxidative stress.^7^ ROS are generally thought to cause deleterious oxidative damage to proteins,^8^ lipids^9^ and DNA.^10^ Polyunsaturated fatty acid residues of lipids in LDL are oxidized by ROS-mediated lipid peroxidation, and the subsequent radical reactions result in the formation of both protein and lipid hydroperoxide on LDL.^11^ *In vitro* studies have indicated that these radical reactions are accelerated in the presence of Fe^2+^ or adrenaline-FeCl_3_ mixture.^12^

We reported previously that 2 h of arm crank ergometer exercise at 60% VO_2_ max increases plasma oxLDL level in able-bodied subjects, but not in subjects with spinal cord injuries (SCI) between T3 and T12.^13^ The same study also showed significantly higher adrenaline levels in able-bodied subjects than in SCI subjects, indicating a positive correlation between plasma adrenaline and oxLDL levels. The formation of oxLDL is dependent on the oxidant/antioxidant balance. Skeletal muscle cells are considered to be the predominant source of ROS in exercise. SCI subjects generally have lower VO_2_ max than able-bodied subjects due to the smaller mass of exercising muscles. Therefore, the low oxLDL level in these individuals may be associated with low production of ROS during exercise. However, only a few studies have evaluated changes in the oxidant/antioxidant balance in SCI athletes during exercise.

The present study was designed to determine the relationship among exercise, adrenaline, oxidant/antioxidant balance and LDL oxidation in athletes with SCI. The study was based on the finding reported by Paulsen *et al.*^14^ that participants with CSCI, but not with lesion of SCI (lower SCI (LSCI)), have impaired sympathetic nervous system function. Specifically, in the present study we measured the levels of derivatives of reactive oxygen metabolites (d-ROMs), a marker of oxidation, and biological antioxidant potential (BAP), as a marker of antioxidation, before and after a wheelchair half marathon. The participants were well-trained athletes with SCI, either cervical SCI (CSCI) or LSCI. We also measured plasma oxLDL and adrenaline levels.

## Materials and methods

### Subjects

Eight subjects with LSCI and nine subjects with CSCI were briefed about the study protocol and possible risks, signed informed consent before the study and voluntarily participated in the present study. Age was 54.9±3.6 (34–65) and 37.7±4.6 (20–64) years (mean±s.e.m. (range)), body weight, 63.0±2.3 (52–72) and 56.4±3.9 (42–83) kg, for LSCI and CSCI athletes, respectively. The lesion levels were T8-L1 and C5-8 for LSCI and CSCI, respectively (Table 1). All subjects participated in the half marathon division of the 34th Oita International Wheelchair Marathon Race in Japan. All subjects participated in a regular physical training before the race and completed the race. Selection criteria used in the present study were the following: (1) males. Female athletes were excluded to avoid possible influence of menstrual cycle-related hormonal changes on the cardiovascular, endocrine and fluid regulation system. (2) Over 1-year post SCI. (3) American Spinal Injury Association (ASIA) Impairment Scale A. (4) Free from acute infection and healthy except for SCI-related dysfunctions. None of the subjects was on any medications that would affect the cardiovascular and endocrine responses during the study period.

The Human Research Committee of Wakayama Medical University School of Medicine approved the present protocol.

### Study protocol

Blood samples were collected from the antecubital vein in the afternoon (between 1200 and 1530 hours) of the day before the race, immediately after the race within 10 min of each racer’s finishing time (1100 to 1200 hours) and 1 h following the completion of the race. Each blood sample was stored into a chilled vacutainer containing ethylenediaminetetraacetic acid (EDTA)-2K for the measurement of blood cell counts and plasma adrenaline and into a lithium heparin-treated syringe for the measurements of oxLDL, d-ROMs and BAP levels.

### Assays of plasma levels of LDL, oxLDL, d-ROMs, BAP and adrenaline

Blood samples were processed immediately for the determination of hematocrit and hemoglobin. Hematocrit and hemoglobin were measured using a full-automatic blood cell counting device. Blood samples were centrifuged at 4 °C immediately after collection, and plasma was separated and the samples were stored at −80 °C in a freezer until analysis. Plasma LDL-cholesterol concentration (LDL-c) was measured using direct narration, and oxLDL was measured using an enzyme-linked immunosorbent assay, based on the principles reported previously by Kotani *et al.*^15^ The d-ROMs test was used for the determination of oxidative stress using plasma samples. This method allows the estimation of the total amount of hydroperoxide present in a 20 μl sample. The results of d-ROMs were expressed in arbitrary units called ‘Carratelli units’ (U.Carr). The antioxidant abilities of plasma were measured by the BAP test. The underlying principle of this test is similar to that of the well-known FRAP test, which measures the ferric-reducing ability of plasma. The test uses a 15-μl sample in a specially designed photometer in conjunction with the FRAS4 system (Wismerll Co., Tokyo, Japan). Adrenaline was extracted from plasma using alumina and measured by high-performance liquid chromatography using a modification of the procedure described by Hunter *et al.*^16^ All measurements were performed in duplicates and completed within 1 month after each sampling.

### Statistical analysis

Data were expressed as mean±standard error of the mean (s.e.m.). Data were analyzed by using one-way analysis of variance, and the difference in variables between CSCI and LSCI at baseline was determined with Student’s *t*-test using GraphPad Prism software (version 5.0; GraphPad Software, Inc., La Jolla, CA, USA) followed by Tukey *post hoc* test. A *P*-value less than 0.05 denoted the presence of a significant difference.

## Results

The characteristics of the participating subjects are presented in Table 1. The LSCI subjects were older than CSCI subjects, and the time since SCI was longer in LSCI subjects. Although height was similar between the groups, the body weight of CSCI subjects (55.8±3.5 kg) was significantly lower than that of LSCI subjects (63.0±2.9 kg). The mean record for the half marathon race was 1:02:30 and 1:26:43 for LSCI and CSCI athletes, respectively.

### Changes in oxLDL level in CSCI and LSCI athletes

**Figure 1:**
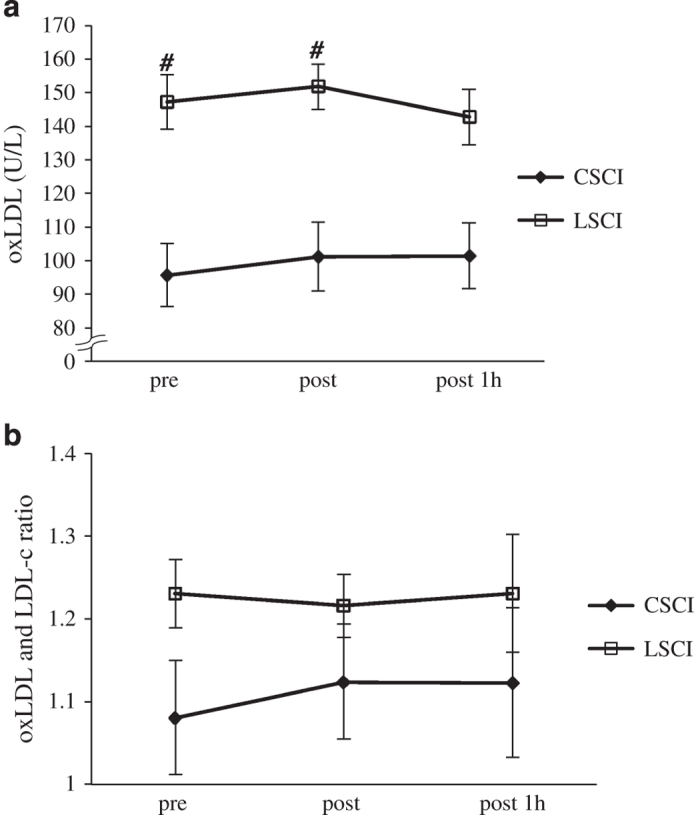
Levels of oxLDL (**a**) and oxLDL/LDL-c ratio (**b**), before (pre), immediately after (post) and 1 h after the half marathon race (post 1 h) in athletes with cervical spinal cord injury (CSCI, *n*=8) and athletes with lower lesion of spinal cord injury (LSCI, *n*=9). ^#^*P*<0.05 vs CSCI.

In LCSI subjects, the oxLDL level before the race was 147.25±8.11 U l^−1^ and did not increase throughout the study. (Figure 1a). Similarly, the oxLDL level before the race was 97.0±10.4 U l^−1^ in CSCI and did not change the levels from pre-race levels versus those assessed immediately after the race. However, the levels before and immediately after the race were significantly higher in LSCI subjects than in CSCI subjects.

LDL-c concentrations were 92.3±9.9, 94.7±10.9 and 94.2±12.0 U l^−1^ before, immediately after and 1 h after the race, respectively, in CSCI athletes, and 120.5±7.5, 125.8±6.9 and 117.4±7.1 U l^−1^, respectively, in LSCI athletes. Thus, LDL-c levels before and immediately after the race were significantly higher in LSCI athletes than in CSCI athletes. The ratio of oxLDL to LDL-c (oxLDL/LDL-c) did not change throughout the study in both groups (Figure 1b). Furthermore, there were no significant differences in the ratio between CSCI and LSCI athletes at any time point.

### Effects of exercise on d-ROMs and BAP levels in CSCI and LSCI athletes

**Figure 2:**
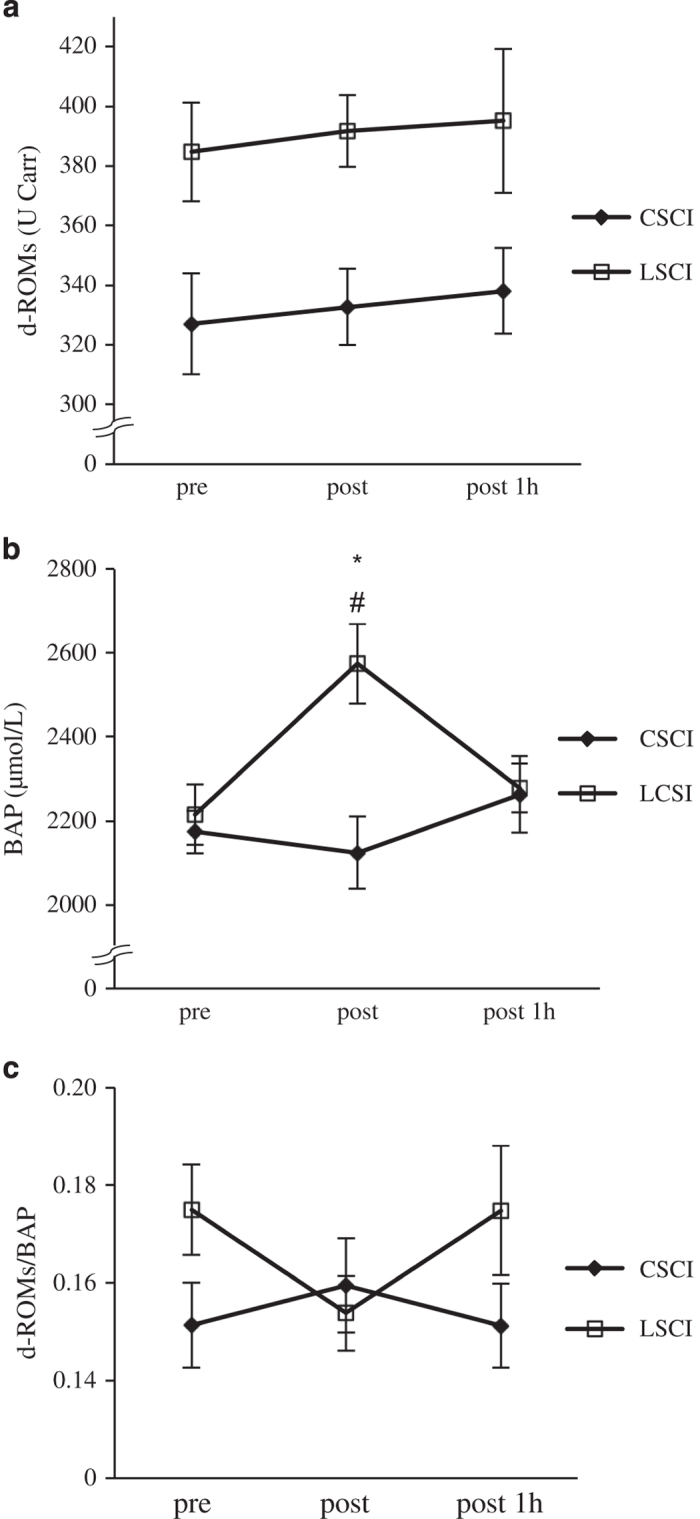
Levels of d-ROMs (**a**), BAP (**b**) and OSI (d-ROMs/BAP) (**c**), before (pre), immediately after (post) and 1 h after the half marathon race (post 1 h) in athletes with cervical spinal cord injury (CSCI, *n*=8) and athletes with lower lesion of spinal cord injury (LSCI, *n*=9). *P<0.05 vs pre; ^#^*P*<0.05 vs CSCI. OSI, Oxidative Stress Index.

Figure 2a shows plasma d-ROMs levels before, immediately after and 1 h after the race. The baseline level of d-ROMs before the race was 329.0±18.7 and 384.6±16.5 U.CARR in CSCI and LSCI, respectively. The d-ROMs levels in both groups were higher than the normal level reported elsewhere (range: 250–300 U.CARR).^17^ The levels in both groups did not change significantly throughout the study. Although the levels in CSCI subjects tended to be lower than those in LSCI subjects at all measurement points, values were not significantly different between the two groups.

In contrast, BAP values increased in response to exercise only in LSCI subjects and were significantly higher than in CSCI subjects (‘post’ in Figure 2b). However, except for this time point, there was no significant difference in BAP values between CSCI and LSCI subjects. Changes in oxidative stress marker (d-ROMs/BAP; Oxidative Stress Index) are illustrated in Figure 2c. Oxidative Stress Index did not change over the sampling period in both groups, and there were no significant differences between the two groups.

### Exercise-induced changes in adrenaline levels in CSCI and LSCI athletes

**Figure 3:**
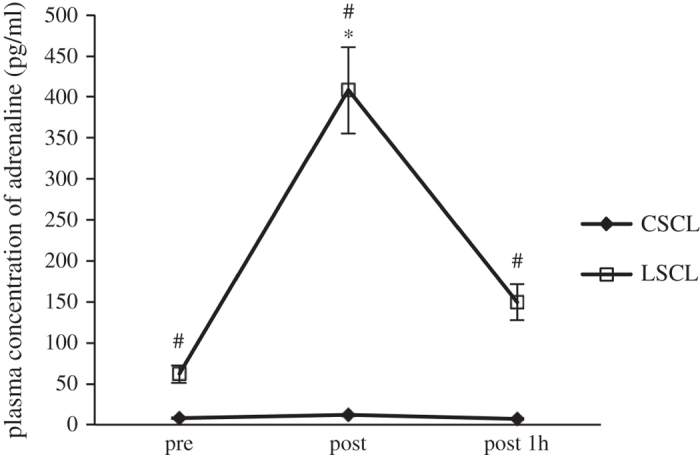
Plasma adrenaline concentrations before (pre), immediately after (post) and 1 h after the half marathon race (post 1 h) in athletes with cervical spinal cord injury (CSCI, *n*=8) and athletes with lower lesion of spinal cord injury (LSCI, *n*=9). *P<0.05 vs pre; ^#^*P*<0.05 vs CSCI.

Figure 3 shows the mean plasma concentrations of adrenaline in CSCI and LSCI athletes before, immediately after and 1 h after the race. Adrenaline concentration before the race was higher in LSCI athletes (62.3±10.8 pg ml^−1^) than in CSCI athletes (8.6±1.2 pg ml^−1^) (*P*<0.05). The level in LSCI athletes increased to 408.0±52.7 pg ml^−1^ (*P*<0.01) immediately after the race, but decreased to 150.0±22.0 pg ml^−1^ at 1 h after the race. In contrast, adrenaline levels in CSCI athletes were 12.3±1.5 and 7.5±0.8 pg ml^−1^ immediately after and 1 h after the race, respectively. The post-race level was significantly lower in CSCI than in LSCI athletes.

## Discussion

We reported previously that the oxLDL level increased after 2-h of arm crank ergometer exercise in able-bodied subjects, but not in subjects with SCI between T3 and T12.^13^ We also demonstrated that the extent of increase in adrenaline in SCI subjects was significantly lower than in able-bodied subjects in that study. Since Fe^2+^ or adrenaline–FeCl_3_ mixture is important for augmentation of oxLDL formation,^12^ we hypothesized that the lack of effect of exercise on the oxLDL level in SCI subjects was due to the lower availability of adrenaline. To confirm this hypothesis, our study determined oxLDL levels before, immediately after and 1 h after the wheelchair half marathon in subjects with LSCI (lesion at T8–L1) and those with CSCI subjects (lesion at C5–8). According to the above hypothesis, LSCI athletes, but not CSCI athletes, are expected to show an increased oxLDL level after a marathon, because a complete SCI above T6 is reported to result in low circulating adrenaline plasma concentrations due to dysfunction of the sympathetic nervous system.^14,18^ However, oxLDL levels remained stable during and after the race in both groups of subjects, although adrenaline increased immediately after the race in LSCI subjects, but not in CSCI subjects. These findings suggest that adrenaline plays only a minor role in the oxidation process of LDL during exercise.

It is well known that oxLDL levels are higher in obese and overweight individuals and correlate with body mass index (BMI) in able-bodied subjects.^19^ OxLDL is also known to be associated with physical activity in SCI:^20^ subjects with chronic SCI exhibited higher oxLDL levels compared with physically active SCI and able-bodied persons. In the present study, we showed that oxLDL levels were higher in LSCI than in CSCI athletes. Since oxLDL levels correlate strongly with those of LDL-c,^21,22^ the higher oxLDL in LSCI athletes could be mainly due to higher LDL-c. Indeed, LDL-c levels in LSCI athletes were higher than in CSCI athletes at rest (*P*=0.04), and the oxLDL/LDL-c ratio was similar in the two groups (*P*=0.09). These results suggest that the higher LDL-c level in LSCI athletes mainly contributes to the higher oxLDL. Furthermore, this higher level of oxLDL in LSCI athletes could be related to older age and higher BMI, since LSCI athletes were significantly older and had higher BMI (*P*<0.05) than CSCI athletes.

It is generally known that aerobic and anaerobic exercises, both maximal and submaximal, stimulate ROS production and elicit oxidative stress.^23^ ROS are important factors in triggering the formation of oxLDL. Thus, in the present study, we measured d-ROMs and BAP as markers of oxidative stress before and after a wheelchair half marathon race. Our results showed no significant changes in d-ROMs and BAP in both LSCI and CSCI athletes during/following the race, except that BAP value increased immediately after exercise in LSCI subjects. Parker *et al.*^24^ reported that intense exercise (>70% VO_2_max) did not increase d-ROMs, but increased BAP levels, indicating that moderate-to-high-intensity exercise significantly increases endogenous antioxidant defenses, possibly to counteract increased levels of exercise-induced ROS. Previous studies reported significantly lower maximum heart rate and VO_2_max in subjects with CSCI than with LSCI.^14,25^ Thus, the exercise intensity of a wheelchair half marathon would be much lower in CSCI athletes than in LSCI athletes. Unfortunately, both the heart rate and VO_2_max were not measured in the present study. Indeed, the recorded time for the half marathon race was more than 20 min longer in CSCI than in LSCI athletes. Thus, it is likely that the increase in the BAP value immediately after exercise in LSCI subjects was to counteract the high levels of exercise-induced ROS. Consequently, not only CSCI athletes but also LSCI athletes could maintain a redox balance during half marathon race, although Oxidative Stress Index tended to decrease after half marathon race in LSCI athletes (*P*=0.09). LDL oxidation was probably unaffected by exercise in both CSCI and LSCI groups due to the maintenance of redox balance.

The antioxidant activity of adrenaline was analyzed in a previous *in vitro* study.^26^ Adrenaline can induce the generation of superoxide anions, with subsequent upregulation of endogenous antioxidant species superoxide dismutase. Moreover, catecholamine secretion parallels the secretion of ascorbic acid from the adrenal glands.^27^ Hence, the increase in BAP observed in LSCI athletes immediately after the race could be associated with enhanced plasma adrenaline release during exercise. Exercise is also followed by increased levels of noradrenaline, which correlate with the increase in certain antioxidants after exercise.^28^ Noradrenaline as well as adrenaline levels increased significantly only in LSCI athletes (data not shown). The increase in adrenaline and noradrenaline levels is a possible mechanism underlying the increase of BAP in LSCI after half marathon race.

There is no information on ROS production at rest or after exercise in SCI subjects. Our study is the first to measure d-ROMs and BAP levels in SCI subjects. The baseline levels of d-ROMs before the race were 329±19 and 385±16 U.CARR in CSCI and LSCI, respectively. The normal reference value provided by the assay manufacturer (Wismerll Co., Tokyo, Japan) for d-ROMs is <300 U.CARR. Nojima *et al.*^17^ also reported a mean value of d-ROMs of 287±100 (±2 s.d.) U.CARR in 312 healthy Japanese subjects. Thus, the d-ROMs levels of our SCI subjects seem to be higher than those published for healthy able-bodied subjects. This result is consistent with the finding of increased ROS production in the spinal cord and peripheral tissues after SCI.^29,30^ The value of d-ROMs increases with age in able-bodied subjects.^17^ Furthermore, adipose tissue contributes to systemic lipid peroxides, and d-ROMs decrease after reduction in BMI.^31^ In our study, LSCI athletes were significantly older with greater BMI than CSCI athletes (*P*<0.05). Therefore, d-ROMs levels may be higher in LSCI athletes than in CSCI athletes due to the older age and higher BMI.

In conclusion, d-ROMs and oxLDL did not change after a wheelchair half marathon in both CSCI and LSCI athletes, whereas BAP increased only in LSCI. Since Oxidative Stress Index did not change during the race, the balance between oxidation and antioxidation was maintained, indicating that exercise intensity of the half marathon race is not excessive for SCI subjects.

## Figures and Tables

**Table 1:** Anthropometric data

	*LSCI subjects*	*CSCI subjects*	P-*value*
Number	8	9	
Age (years)	54.9±3.6	37.7±4.6	0.006
Height (cm)	170.1±1.3	171.7±2.8	NS
Weight (kg)	63.0±2.3	56.4±3.9	0.0003
Spinal cord lesion	T8–L1	C5–8	
ASIA	A	A	
Story of injury (year)	29.5±4.9	15.9±3.6	0.03

Abbreviations: ASIA, American Spinal Injury Association; NS, nonsignificant.
